# Are Anti-rhGAA Antibodies a Determinant of Treatment Outcome in Adults with Late-Onset Pompe Disease? A Systematic Review

**DOI:** 10.3390/biom13091414

**Published:** 2023-09-19

**Authors:** Imke A. M. Ditters, Harmke A. van Kooten, Nadine A. M. E. van der Beek, Ans T. van der Ploeg, Hidde H. Huidekoper, Johanna M. P. van den Hout

**Affiliations:** 1 Department of Pediatrics, Center for Lysosomal and Metabolic Diseases, Erasmus MC, Erasmus University Medical Center, P.O. Box 2060, 3000 CB Rotterdam, The Netherlands; 2 Department of Neurology, Center for Lysosomal and Metabolic Diseases, Erasmus MC, Erasmus University Medical Center, P.O. Box 2060, 3000 CB Rotterdam, The Netherlands

**Keywords:** late-onset Pompe disease, anti-rhGAA antibodies, enzyme replacement therapy, systematic review

## Abstract

Background: Pompe disease is a lysosomal storage disease characterised by skeletal and respiratory muscle weakness. Since 2006, enzyme replacement therapy (ERT) with alglucosidase alfa has been available. ERT significantly improves the prognosis of patients with Pompe disease. The effect of high antibody titres on treatment response in adults with late-onset Pompe disease (LOPD) remains unclear but may contribute to interpatient variation. We therefore conducted a systematic review on this subject. Methods: A systematic search was performed in Embase, Medline Ovid, Web of Science, Psych Info Ovid, Cochrane (Clinical Trials only), and Google Scholar (random top-200). Articles were included if they involved adults with LOPD treated with alglucosidase alfa and mentioned anti-rhGAA antibodies or antibody titres. In addition, articles mentioning dosages different from the standard recommended dosage were included. Results: Our literature search retrieved 2562 publications, and 17 fulfilled our selection criteria, describing 443 cases. Seven publications reported on anti-rhGAA antibody titres on a group level, with the percentage of patients with a high titre as defined in the included articles ranging from 0–33%. Six publications reported on the effect of anti-rhGAA antibody titre on clinical course, and four found no correlation. Two studies reported a negative effect on treatment. The first study found a greater improvement in Medical Research Council (MRC) score in patients with no detectable antibody titre. In the second study, a patient discontinued ERT due to a declining neuromuscular state as a result of high anti-rhGAA antibody titres. Seven publications reported on 17 individual patients with a high antibody titre (range 1:12,800–1:3,906,250). In only two cases were high-sustained neutralising antibodies reported to interfere with treatment efficacy. Conclusions: No clear effect of anti-rhGAA IgG antibodies on treatment response could be established for the majority of LOPD patients with a high antibody titre. In a minority of patients, a clinical decline related to (possible) interference of anti-rhGAA antibodies was described.

## 1. Introduction

Pompe disease, or glycogen-storage disease type 2 (OMIM #232300), is a progressive lysosomal storage disease with autosomal recessive inheritance. The disease is caused by a deficiency of the enzyme acid α-glucosidase [1]. The clinical spectrum of the disorder is broad and continuous. Patients with classic infantile Pompe disease have a severe enzyme deficiency with virtually no residual activity of α-glucosidase, leading to severe hypotonia and hypertrophic cardiac myopathy with a fast progressive course of disease [1]. Patients with late-onset Pompe disease (LOPD), also known as non-classic Pompe disease, have higher residual enzyme activity levels, albeit usually no more than 20–30% of normal average GAA activity [1]. In these patients, the enzyme deficiency leads to a slowly progressing axial and limb girdle pattern of skeletal as well as respiratory muscle weakness. These patients can have a disease onset at any age and can therefore be divided into a childhood and an adult-onset phenotype. Usually, they do not have cardiac involvement and have a more slowly progressing disease course compared with the classic infantile patients. Ninety percent of LOPD patients with the childhood-onset or adult phenotype have the most common c.-32-13T>G variant [2,3,4]. Since 2006, enzyme replacement therapy (ERT) with recombinant human alpha-glucosidase ((rhGAA) alglucosidase alfa (Myozyme^®^)) has been available [1]. New ERTs such as avalglucosidase alfa and cipaglucosidase alfa/miglustat have been approved for the treatment of patients with LOPD in the last two years [5,6].

In patients with LOPD, treatment with alglucosidase alfa is administered once every two weeks [7]. ERT significantly slows down disease progression in most adult Pompe disease patients, leading to improvement or stabilisation of respiratory function as well as skeletal muscle strength and function; ERT also has positive effects on daily life activities, [3,7,8,9,10,11,12,13,14,15] with a peak effect at approximately 2–3 years following start of treatment [2]. Despite these beneficial effects of ERT, a considerable individual variation in response to ERT is seen. The majority of patients (83%) experience a slow, seemingly linear secondary clinical decline after approximately 3–5 years of treatment [16]. It is difficult to predict the occurrence and timing of a change in responsiveness to ERT in adults with Pompe disease [16]. In classic infantile patients, one of the factors negatively influencing treatment response is the occurrence of anti-rhGAA antibodies [17]. This applies mostly to Cross Reactive Immunologic Material (CRIM)-negative patients, who do not produce any residual endogenous alpha-glucosidase, but also to CRIM-positive patients, who do produce some non-functional alpha-glucosidase [18,19]. This has led to the implementation of immunomodulation strategies to mitigate the effects of anti-rhGAA antibodies [20,21,22,23,24,25,26]. Anti-rhGAA antibodies have the potential to interfere with ERT by binding to the infused enzyme and reduce uptake into the cell or inhibit catalytic activity and thus treatment effectivity [27,28]. The effect of neutralizing antibodies can be partially explained by the balance between the antibody titre and the dosage of ERT, with more enzymes reaching the target tissues at higher dosages [18,29,30]. Patients with LOPD have residual enzyme activity and are CRIM-positive by definition. Although multiple publications demonstrate that treatment response can be negatively impacted by antibody formation in classic infantile Pompe patients [17,18,19,31], we recently showed that high antibody titres do not seem to interfere with clinical outcomes in LOPD patients with childhood-onset Pompe [32]. To date, the effect of high antibody titres on treatment response in adults with late-onset Pompe patients remains inconclusive.

Here, we present a systematic review of the occurrence and effects of anti-rhGAA antibody titres on treatment efficacy (e.g., pulmonary function, muscle strength and function), and their relationship with infusion-associated reactions (IARs) in adults with late-onset Pompe disease.

## 2. Methods

### 2.1. Search Strategy

A systematic search was performed in Embase, Medline Ovid, Web of Science, Psych Info Ovid, Cochrane (Clinical Trials only), and Google Scholar (random top-200). The first search was performed on 12 March 2019, and an updated search was executed on 21 June 2022. Each search was conducted using a set list of vocabulary and keywords. There was no language limit. The search was limited to articles involving human subjects and articles published after 1999, when treatment with enzyme replacement therapy for Pompe disease was first administered in patients with Pompe disease on a trial basis. See Supplementary File S1 for specific search terms and search strategy.

### 2.2. Selection Criteria

Articles were included based on the following predefined acceptance criteria: The publication should be addressing adults with LOPD, which was defined as patients with an age at symptom onset of 18 years or older. If the age at symptom onset was not available, the current patient age as described in the publication was used for in- or exclusion of the patient. The patients had to receive treatment with alglucosidase alfa. The title or abstract should mention antibodies or antibody titres. Articles were also included if they mentioned an ERT treatment dosage other than the standard recommended dosage of 20 mg/kg/every other week, as this may influence antibody formation. Randomised controlled trials, observational studies, cohort studies, case-control studies, cross-sectional studies, case reports, and case series were considered eligible for inclusion. Reviews were excluded.

### 2.3. Selection Strategy

Two reviewers (ID and HvK) performed title and abstract screening. Subsequently, articles were screened based on their full texts. Thereafter, discrepancies in the selection of publications between the two reviewers were discussed. The remaining uncertainties regarding inclusion were discussed with three other reviewers (JvdH, NvdB and HH). Additional publications were identified by reviewing the references of selected articles as well as by assessing the full text of the included publications and selecting possibly relevant publications based on the paraphrased or quoted source text. After the completion of the manuscript, an updated literature search was performed on 21 June 2022. Three additional articles were found and were reviewed using the same selection strategy.

### 2.4. Data Extraction and Results

Data was extracted from included publications using a data extraction table. General data on the study characteristics comprised author, year of publication, title, study design, sample size, and study outcomes. At a group level, the following data were collected: sex, age (mean/median), age at onset (mean/median), age at diagnosis (mean/median), treatment dosage, treatment duration, % of patients with a high antibody titre (as defined in the included articles), antibody titre range, neutralising effect of antibodies, definition of high sustained antibody titres (HSATs), % of patient IARs, and treatment response. On an individual patient level, the following data were collected: sex, age, *GAA* disease-causing variants and corresponding protein changes, age at onset, age at diagnosis, age at start of treatment, dosing, treatment duration, highest antibody titre and neutralising effect (the effect of antibodies on the uptake of rhGAA), the definition of high antibody titer and reported HSAT per study, IARs, and treatment response.

Data extraction was performed by two reviewers (ID and HvK) and then compared to ensure the reliability and accuracy of the extracted information. Discrepancies and remaining uncertainties were discussed with three other reviewers (HvdH, NvdB and HH).

## 3. Results

### 3.1. Study Selection

**Figure 1:**
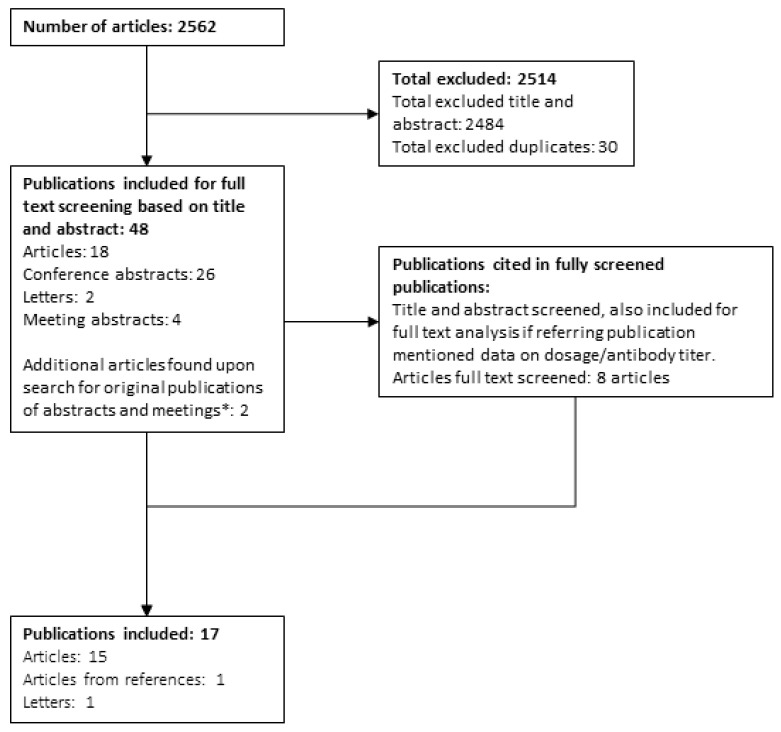
Study selection flow diagram (adapted from the Preferred Reporting Items for Systematic Reviews and Meta-Analyses (PRISMA) template) [46]. * Not included in total of 2562 articles.

Our literature search retrieved 2562 publications in total: 1883 publications from the first search and 679 from the second search. For all conference and meeting abstracts—26 in total—the availability of the original publication was checked, and two additional full publications were found. For one of these publications, the abstract of the final publication did not mention either anti-rhGAA antibodies or dosing, whereas the conference abstract did. Another publication was published after the initial search, but prior to the updated search, and was included based on the conference abstract. In addition, we checked the reference lists of included publications and identified one additional publication that could be included. In the end, 17 publications fulfilled our selection criteria and were thus included in the review (Figure 1) [3,5,7,29,33,34,35,36,37,38,39,40,41,42,43,44,45]. Three publications were included from the updated search on 21 June 2022. In total, these papers described 443 cases, with some overlap between papers.

### 3.2. Study Characteristics

The characteristics of the included publications (*n* = 17) are summarised in Table 1 [3,5,7,29,33,34,35,36,37,38,39,40,41,42,43,44,45]. Included publications were: randomised controlled trials (*n* = 2) [2,41], case reports (*n* = 5) [29,33,34,36,39], and cohort studies (*n* = 10) [3,35,37,38,40,41,42,43,44,45]. Two publications had an overlap of at least one patient [29,41]. Please note that some articles had antibody titres as an inclusion criterion, and thus not as a study outcome.

Several publications reported on the main parameters of interest for this study, anti-rhGAA antibody titres, and/or dosing at a group level [3,5,7,38,40,42]; others reported this on a patient level only [29,33,34,36,37,39,43]. Four publications reported on these parameters on both a patient level and group level [35,41,44,45]. Hence, we have split the data into group level data (Table 2) and patient level data of patients with a high antibody titre (Table 3). Data on individual patients who did not have a high antibody titre are described in Supplementary Table S1.

### 3.3. Definition of High Antibody Titre and High Sustained Antibody Titre (HSAT)

The publications included in this review had different definitions of a high antibody titre and/or a HSAT; in addition, the time point(s) at which antibody titres were measured differed between studies. Of the 10 publications [3,5,7,35,38,40,41,42,44,45] that reported data on a group level, seven publications gave their definition of a high titre [5,7,35,38,41,42,45], with the definition of a high titre ranging from ≥12,800 [5], 25,600–819,200 [7], ≥1:31,200 [41], ≥1:31,250 [45], >1:31,250 [42], to ≥1:51,200 [35,38]. Eleven publications reported data on an individual patient level [29,33,34,35,36,37,39,41,43,44,45]; five of these publications gave a clear definition of a high titre [35,37,41,43,45], with the definition of a high titre ranging from ≥1:31,200 [41], ≥1:31,250 [45], >1:31,250 [43], to ≥1:51,200 [35,37]. A high sustained titre was defined in two publications which reported on data on a group level [35,38] and two publications which reported on an individual level [35,37]. A HSAT was defined as a titre of ≥1:51,200 on two or more occasions at or beyond 6 months on ERT [35], ≥1:51,200 on more than two occasions at or beyond six months on ERT [38], or >1:51,200 on two or more occasions at or beyond 6 months on ERT [37]. Since the definition of what constitutes a high antibody titre varies between publications, any generalised reference to high antibody titres in this review corresponds to the definition of a high antibody titre as provided in the respective publications.

### 3.4. Data on a Group Level

Data from 10 publications [3,5,7,35,38,40,41,42,44,45] reporting on anti-rhGAA antibody titres and outcomes on a group level are summarised in Table 2. Between 0% and 33% of patients in these studies had a high antibody titre (group size *n* = 4 to *n* = 73). Absolute antibody titres in these patients were reported in seven publications with a population size ranging from *n* = 6 to *n* = 73 [3,5,7,40,41,42,45]. Antibody titres ranged from no detectable titre to 1:819,200. Eight publications [3,5,7,35,38,41,44,45] reported on IARs in their patient population, with the number of patients with IARs varying from none (*n* = 0/6) [45] to 33% of patients (*n* = 16/49) [5]. Two studies assessed if there was an association between serum immunoglobulin G (IgG) antibody titre and the occurrence of IARs on a group level. The first of these two studies, which tested anti-rhGAA antibodies in 59 patients over an 18-month follow-up period, found no association between anti-rhGAA titre and IARs [7]. Antibody titres in this study ranged from 1:200–1:819,200 [7]. The other study, with a study population of 73 patients and a median follow-up duration on ERT of 40 months (range, 24–42 months), found that the height of the titre positively correlated with the occurrence and number of IARs [41]. In the latter study, only 1/28 (4%) patients in the no-to-low-titre group experienced IARs, 5/29 (17%) in the intermediate-titre group experienced IARs, and 7/16 (44%) in the high-titre group experienced IARs (*p* = 0.001) [41]. A neutralisation assay was performed in the 16 patients in this study with a high titre; eight of these patients had a maximum titre of 1:31,250 and neutralising effects from 0–15%, and the other eight patients with a maximum titre of ≥1:156,250 had a neutralising effect ranging from 15–85% and are described in more detail on a patient level below [41].

Nine [3,5,7,38,40,41,42,44,45] out of 10 publications reported on patient outcomes on a group level. Six of these publications also assessed the effect of anti-rhGAA antibody titre on clinical course [3,7,40,41,42,45]. Four studies did not find an effect of antibody titre on treatment outcome [7,40,41,45]. In two of these studies, no correlation was found between anti-rhGAA antibody titre and clinical endpoints [7,40], although in one study a subset of LOPD patients who received long-term ERT had elevated levels of anti-rhGAA IgG1 and IgG4 antibodies, whereas no significant levels of IgG2 and IgG3 were detected [40]. A third study [41] found that in all three titre groups (i.e., no-or-low, intermediate, high antibody titre), patients with an improved, stable, and declining clinical course were present, and concluded that antibody formation does not interfere with rhGAA efficacy in the majority of adults with Pompe disease. The fourth study [45] found that there was no association between anti-rhGAA antibody titres and clinical outcomes. In fact, in this study, the patients with the best clinical results under treatments also showed the highest IgG antibody titres against rhGAA (up to 1:102,400). Two studies did find an effect of anti-rhGAA antibody titre on clinical outcomes [3,42]. The first study [42] noted that, in the subgroup treated for less than 36 months, patients with no detectable antibody titre (21.9%) showed a significantly greater (*p* = 0.039) clinical improvement (delta 1.92, SD 1.32) in MRC sum score than those with detectable antibodies (delta −0.37, SD 0.81). Of the patients with a positive titre, 31.2% had a low titre (<1:1250), 43.8% a medium titre (1:1250–1:31,250), and 3.1% a high titre (>1:31,250). In the second study, one patient was reported to have discontinued ERT due to a declining neuromuscular state because of high anti-rhGAA antibody titres (1:819,200) [3].

### 3.5. Data on a Patient Level

Data from eleven publications [29,33,34,35,36,37,39,41,43,44,45] reporting on 17 (23%) individual patients with high anti-rhGAA antibody titres are summarised in Table 3. The remaining 56 patients (77%) with low-to-intermediate antibody titres can be found in Supplementary Table S1.

### 3.6. Patients with a High Antibody Titre

Seven publications reported on 17 patients with a high antibody titre (Table 3) [29,34,35,41,43,44,45], 23% of patients in whom the antibody titre was reported on a patient level. Titres varied from 1:12,800 to 1:3,906,250 [29,34,35,41,43,44,45]. For one patient, the exact titre was not reported [44]. In 10 patients (one of whom was described in two studies and is thus mentioned in the table twice [29,41]), a neutralisation assay was performed, measuring either GAA enzyme activity in the patient’s serum or uptake of rhGAA by cultured human fibroblasts [34,41]; 2 had no neutralising antibodies [34,41], 3 patients experienced a temporary neutralising effect which resolved over time, and 4 patients were described as having experienced strong neutralising effects [41]. In the tenth patient, an immunologic assay performed at an antibody titre of 1:800,000 demonstrated that 42% of the administered alglucosidase alfa was captured by circulating antibodies. In this same patient, cellular uptake of alglucosidase alfa in fibroblasts was also inhibited [29]. A second study demonstrated strong neutralising effects measured in vitro in fibroblasts, at a titre of 1:3,906,250 in the same patient after longer follow-up, with concomitant declining MRC sum scores and FVC scores [41]. In this patient, ERT was eventually discontinued. The clinical course was reported for 14/17 patients and was thus not reported for three patients [34,44]. Nine patients did not experience a clinical decline on ERT. In four patients, antibodies were reported not to affect treatment response [41]. A fifth patient improved or stabilised on all parameters but FVC [45]. In three patients, the treatment response was unclear due to high, stable MRC scores and FVC scores [41]. The ninth patient had muscle function tests and spirometry that remained stable after one year of progression [43]. In total, 5 of the 14 patients (36%) in whom both antibody titre and the clinical course were reported experienced an overall clinical decline [29,35,41]. The *GAA* disease-causing variants were provided for 9 of the 17 patients with high antibody titres, 8 of whom carried the most common c.-32-13T>G (IVS1) disease-causing variant. The second variant differed between these patients, but two siblings both had the c.2655_2656delCG variant [44]. One patient did not carry the c.-32-13T>G variant but carried the c.1781G>A and c.1194+5G>A variants instead [43]. No correlation between the *GAA* variant and the development of high antibody titres could be established.

### 3.7. Patients with a No-to-Intermediate Antibody Titre

In eight publications [29,33,36,37,39,43,44,45], 56 patients with no-to-intermediate antibody titres were reported, 77% of patients in whom the antibody titre was reported on a patient level. (Supplementary Table S1). Antibody titres varied between no anti-rhGAA antibodies to a titre of 1:25,600. For 12 patients, the exact antibody titre was not reported [36,44] and neutralisation assays were not performed. Five of these publications reported on patients’ treatment response to alglucosidase alfa infusions [29,36,37,39,45]. Of the 20 patients with no-to-intermediate antibody titres included in these publications, no apparent effect of antibodies was reported in 14 patients. Three patients had a good response to ERT [29]. In the fourth patient, ERT had significant efficiency regarding improvements in clinical and respiratory tests [39]. In the fifth patient, it was reported that the antibody titre did not seem to impact functional status [37]. In the sixth patient, the treatment showed a significant effect regarding the improvement of clinical tests and respiratory investigations [39]. Finally, eight patients had a stable disease course under ERT [37]. A decline in outcome of therapy was reported in six patients. One patient clinically deteriorated at 40 months of ERT, with a need for non-invasive ventilation and walking aids [37]. Five elderly patients all declined on most parameters [45], despite low-to-intermediate antibody titres. The *GAA* disease-causing variants were known for all but two patients, but for five patients only the variant on one allele was reported. Forty-seven of the 56 patients (83.9%) had the c.-32-13T>G variant on one allele.

### 3.8. IARs and Antibody Titres

Six publications reported on IARs in 28 patients on an individual patient level [29,33,35,39,44,45].

Twenty-one of these 28 patients had a no-to-intermediate antibody titre and two out of these 21 patients experienced IARs [33,39], one had an intermediate antibody titre of 1:1600 [33], and in one patient no antibodies were detected [39]. For one of these two patients, a clinical response to ERT was reported, with this patient showing a significant efficiency of ERT regarding improvement in clinical tests and respiratory investigations [39]. A total of 7 of the 28 patients in these publications had a high antibody titre, with titres ranging from 1:12,800 to 1:819,200. Four of these seven patients experienced ≥one IAR during follow-up, while one patient was described as not experiencing any significant IARs and two patients did not experience any IARs. Of the four patients who experienced ≥one IAR during follow-up, the effect of ERT was not reported in one patient [44]. In the mentioned study [44], the only reported IAR coincided with an elevated antibody titre of 1:12,800. Two patients showed an initial stabilising effect or even clinical improvement under ERT [35], and one patient clinically declined on ERT [29]. Eventually, all three patients with one or more IARs during follow-up and high antibody titres for whom clinical status was reported experienced a clinical decline despite ERT [29,31].

### 3.9. IgE and IgM Antibodies

IgE antibody titres were measured in five publications [7,33,38,39,40]. In one study, three patients (5%) experienced anaphylactic reactions, two of whom tested positive for anti-rhGAA IgE antibodies [7]. Another study reported a patient with a history of anaphylaxis to alglucosidase alfa who had a positive IgE antibody test once [33]. In this patient [33], skin-prick and complement testing were negative and tryptase levels were normal at weeks 9 and 38, respectively. The height of the IgE antibody titres was not reported in either of these studies. A third study screened 28 patients on IgE and IgM antibodies. In none of these patients, a significant level of IgE or IgM was detected [40]. The other two studies either reported on a patient who had a negative anti-rhGAA IgE test in blood, with skin-prick tests being negative as well despite IARs and an anaphylactic reaction to ERT, [39] or the outcomes of the test were not reported [38].

### 3.10. ERT Dosing and Antibody Formation

Only three publications reported on dosages other than the standard recommended dosage of 20 mg/kg/eow [33,36,38]. A dosage higher than the standard recommended dosage was prescribed in one study to compare alternative dosages in patients with a clinical decline or lack of improvement on the standard dosage [38]. In the publication with a dosage increase [38], outcomes were reported on a group level in patients with declining motor function. Two patients received a dosage of 20 mg/kg/week and two patients received a dosage of 40 mg/kg/eow [38]. Two of these patients (one receiving 20 mg/kg/week and one receiving 40 mg/kg/eow) showed improvement on a higher dosage. Of the other two late-onset patients in this study, one (receiving 40 mg/kg/eow) showed no significant change in motor function and the other one (receiving 20 mg/kg/week) died [38]. None of these patients developed a high antibody titre.

The reasons to prescribe a dosage lower than the standard recommended dosage were: to improve a patient’s quality of life [36] and for desensitisation purposes [33]. In the first publication with a dosage decrease, the patient was initially treated with 20 mg/kg/eow and later switched to 20 mg/kg/every 4 weeks [36]; this patient remained stable during 40 months of follow-up. Antibody titre was not reported in this patient. In the second publication with a decrease in dosage, the patient was initially treated with a dosage of 20 mg/kg/eow, switched to 10 mg/kg/week, and later switched back to a dosage of 20 mg/kg/eow and back to 10 mg/kg/week due to allergic reactions [33]. Although the patient occasionally developed mild urticaria and still needed premedication of diphenhydramine and prednisone, desensitisation to the enzyme, partially by lowering the dosage of ERT, ameliorated the IARs and allowed treatment with ERT to proceed in this patient [33]. The maximal antibody titre in this patient was 1:1600 [33]; see Supplementary Table S1.

## 4. Discussion

We conducted a systematic review of the effect of anti-rhGAA antibodies on treatment outcomes in adults with LOPD. In total, 17 publications were included, which described 443 cases. Overall, 23% of individually described patients developed a high titre. Based on our results, no clear effect of anti-rhGAA IgG antibodies on treatment response could be established for the majority of adult Pompe disease patients with a high antibody titre.

On a group level, two studies [7,40] found no correlation between the height of the anti-rhGAA antibody titre and clinical endpoints. One publication [42] reported that, in their subgroup treated for less than 36 months, patients with an undetectable antibody titre showed a greater clinical improvement in MRC sum score than those with a positive antibody titre. On an individual patient level, only 17 individual cases with high antibody titres were reported. A total of 5 of the 14 patients in which the clinical course was described exhibited a clinical decline during treatment (Table 3), whereas 6 of the 20 patients with no to an intermediate antibody titre for whom clinical status was reported showed a clinical decline despite ERT. Five of these six patients were elderly and the clinical decline could be partially attributed to their age. Overall, based on this systematic review, the development of high antibody titres cannot not unequivocally be related to a clinical decline or decrease in the effect of ERT in adults with late-onset Pompe disease. Thus, the effect of IgG antibodies on the efficacy of ERT remains under debate for LOPD [29,35,40,41,42,45], with anti-rhGAA IgG antibodies not significantly impacting clinical outcomes in the majority of LOPD patients [40,41,45]. However, clinical outcome was reported to be affected by high anti-rhGAA titres in some individual patients [29,35]. Exemplary is one study [38], in which, of the eight patients that developed very high maximal titres of ≥156,250, only one patient had high sustained neutralising antibody titres that probably interfered with treatment efficacy. We would expect a similar pattern in newer enzyme replacement therapies; this is indeed what was seen in a study comparing cipaglucosidase alfa/miglustat to alglucosidase alfa/placebo, with treatment effects not influenced by the presence of antidrug antibodies in a cohort of LOPD patients [47]. Antidrug antibody data indicate that avalglucosidase alfa is not more immunogenic than alglucosidase alfa. Fewer participants in the avalglucosidase group compared with the alglucosidase alfa group developed high peak titres and neutralising antibody responses were more commonly reported in the latter group [5].

IgG-mediated immune reactions seem to be the most prevalent immune response to ERT in LOPD, with the LOTS study reporting seroconversion in all patients in their cohort [7]. However, no significant association between serum IgG antibody levels and the effect of ERT or incidence of IARs was found in this study [7]. High IgG antibody titres may lead to lower bioavailability of ERT, by binding catalytically active enzymes, and may hamper uptake by the target tissue as demonstrated in the patient with the highest titre in the paper by de Vries [29]. Depending on their binding sites, antibodies can block binding to the mannose-6-phosphate receptor and prevent uptake of the enzyme, inhibit catalytic function, or misdirect the enzyme to macrophages and neutrophils [28,48].

In 10 of the 17 patients with high titres, neutralising assays—analysing the neutralising effect of anti-rhGAA antibodies—were performed. In three patients, a temporary neutralising effect was found, while in four patients a strong neutralising effect was found, in vitro [41], with clear interference of anti-rhGAA IgG antibodies with ERT efficacy observed in two cases. In one of these patients, 42% of the administered alglucosidase alfa was captured by circulating antibodies, meaning that only about half the dosage of alglucosidase alfa was available for cellular uptake, which has been demonstrated to be insufficient to fully clear glycogen from skeletal muscle tissue [49,50,51,52]. Indeed, in this patient, a decline in clinical parameters whilst on ERT was seen. However, this was not the case for all patients with neutralising anti-rhGAA antibodies, implying that the in vitro neutralising activity of anti-rhGAA antibodies does not always correlate with a clinically relevant effect of anti-rhGAA antibodies on treatment efficacy in adult Pompe patients.

The difference between patients in the clinical effects of anti-rhGAA antibodies may be explained by the different IgG subclasses produced in response to ERT, with IgG4 having been reported in association with the inhibiting activity of ERT in haemophilia A [53]. However, this could not be confirmed in a cohort of LOPD patients as only non-neutralising antibodies, including IgG4, were detected [40].

In addition, IgG antibodies and antigens can form immune complexes, triggering infusion-associated reactions [54]. Overall, it was reported for 28 individually described patients whether they developed IARs. A total of 2 out of 21 patients (9.5%) in the no-to-intermediate titre group developed IARs, compared with 4 out of 7 patients with a high antibody titre (57%). This is in line with one of the articles included in this review, that found on a group level, in a cohort of 73 patients, that the height of the antibody titre positively correlated with the occurrence and number of IARs [41]. However, another study which measured anti-rhGAA antibody titres in 59 patients found no association between anti-rhGAA titre and infusion-associated reactions [7]. Antibody titres in this study ranged from 1:200–1:819,200 [7]. Thus, it is very plausible that an association exists between IARs and antibody titres in adults with LOPD, though more research in large cohorts is needed, as the literature is still inconclusive.

The other type of immune reaction that has been reported in Pompe disease is IgE-mediated [55]. Several publications included in this review reported on IgE antibodies [7,33,38,39] but only three patients across two publications tested positive for IgE [7,33]. Based on these data, the effect of IgE antibodies on treatment outcomes in Pompe disease remains elusive.

Due to the small number of individual patients with high antibody titres, we could not determine if there is a correlation between the *GAA* variant and the development of high antibody titres, which is in line with what was previously reported [40]. Nevertheless, another study suggests that the common combination of variants—c. -32-13T>G (IVS1)/c.2481+102_2646+31del (delex18)—may attenuate antibody formation, with none of the patients with this genotype developing high antibody titres [41]. However, it should be noted that the c.-32-13T>G (IVS1) variant is the most frequently occurring genetic variant in LOPD and is present in about 90 percent of Pompe patients [2,3,4]. This splicing variant leads to the production of some wild-type GAA protein, although only 10 to 15% of transcripts are spliced normally, potentially making the development of high antibody titres less likely [56,57]. Nonetheless, patients with the c.-32-13T>G (IVS1) disease-associated variant can develop high neutralising antibodies [29,41]. It is known that in classic infantile patients CRIM status determines the risk of antibody formation, with 85% of CRIM-negative patients developing HSAT compared with 32% of CRIM-positive patients [31,58]. One could thus speculate that LOPD patients, having a CRIM-negative variant as the second disease-causing variant, would be more prone to developing high antibody titres. However, this does not fully explain variation between patients, suggesting that factors other than genotype contribute to the development of antibodies.

An ERT dosage higher than the standard recommended dose of 20 mg/kg/eow has been shown to improve survival in classic infantile Pompe disease patients [30], but can potentially also make patients more prone to the development of anti-rhGAA IgG antibodies [59]. We, therefore, aimed to assess the effect of ERT dosing on outcome and the development of antibodies in adults with late-onset Pompe disease. Since very few patients were treated with a dosage other than the standard recommended dose of 20 mg/kg/eow, we could not analyse the effect of alternative dosages on disease progression or antibody formation in adults with late-onset Pompe disease.

We identified several limitations in our analysis of the data derived from the included articles: Firstly, different definitions of a high antibody titre as well as a high sustained antibody titre were given, making it difficult to compare study outcomes in this regard. The definition of a high antibody titre ranged from 1:12,800 to ≥1:51,200, which may be partially dependent on the titration steps used. Most papers did not specify what the source was for the reference values for (highly sustained) antibodies. It has been demonstrated in the serum of classic-infantile Pompe patients that there is probably no effect on enzyme activity or uptake of alglucosidase alfa with an antibody titre of ≤1:6250. Alglucosidase alfa activity in the medium was not inhibited until titres of 1:31,250 were reached [18], indicating that it is only useful to measure neutralising antibodies for a titre of ≥1:31,250. This same study also arithmetically estimated that, whereas titres of 1:31,250 may counteract ERT at a dosage of 20 mg/kg, titres above 1:60,000 are expected to counteract ERT when a dosage of 40 mg/kg is administered [18]. These estimates are consistent with the suggested cut-off value for a high titre of 1:51,200 [19] and demonstrate that, to interpret the clinical effect of anti-rhGAA antibodies, the dosage of ERT must be known as well [18]. We would suggest using the following definitions of a high and high sustained antibody titre: a high titre means a titre ≥ 31,250 and a high sustained antibody titre means a titre of ≥31,250 on two or more occasions at or beyond six months of ERT.

Secondly, the level of evidence of the included publications was variable, with 5 out of 17 included papers being case reports, and the outcome of interest (antibody titre and ERT dosage) was often not the main outcome of the paper. As some patients were reported in multiple publications—we are aware of at least one patient for whom this was the case—the number of unique patients described in this review was lower than 443. Multiple publications included patients with different phenotypes which contributed to a larger clinical heterogeneity than intended for our study. Finally, due to the heterogeneity of clinical outcome parameters, the different definitions of high (sustained) antibody titres, different time points at which antibody titres were measured, as well as limited data about dosing, we could not analyse antibodies over time, nor could a meta-analysis be performed. We resolved this by using the definition of a high (sustained) titre as used in the original publication and by looking at the highest antibody titre reported per patient. In addition, it must be noted that there might be a selection bias in that patients with high antibody titres or IARs were more likely to be reported, and that follow-up was not always continuous over time and varied between studies. For future studies, we advise to (1) standardise the definition of a high antibody titre (≥31,250) and of a high sustained antibody titre (high antibody titre on two or more occasions at or beyond six months of ERT) to improve comparability, (2) perform regular, standardised follow-up of antibody titre (with collection of samples prior to ERT infusion), which will provide insight into the development and course of antibody titre over time and prevent sampling bias, as well as (3) carefully describe patient characteristics and standardize clinical follow-up to enable assessment of the effect of antibody formation on outcome of ERT in individual patients.

In conclusion, it appears that anti-rhGAA IgG antibodies do not affect treatment outcomes in the majority of patients, but may have an effect on treatment outcomes in a small subset of adults with LOPD. Further prospective long-term research, aimed at assessing this relation, is required. We suggest that determining anti-rhGAA antibody titres should be part of the standard follow-up in patients with LOPD, especially in case of clinical deterioration or when accompanied by IARs.

## Figures and Tables

**Table 1:** **Overview of included publications and their characteristics.** Immunoglobulin (Ig), recombinant human alpha-glucosidase (rhGAA), Medical Research Council (MRC) sum score, Hand Held Dynamometry (HHD), 6-Minute Walking Test (6MWT), Forced Vital Capacity (FVC), infusion-associated reactions (IARs), 36-Item Short Form Health Survey (SF36), creatine kinase (CK), Walton Gardner Medwin (WGM) scale, Vital Capacity (VC), Gross Motor Function Measure-66 (GMFM-66), Pompe Pediatric Evaluation of Disability Inventory (PEDI), Manual Muscle Test (MMT), Physical Component Summary (PCS), Medical Outcomes Study Short Form-36 (MOS SF-36), adverse events (AEs), electrocardiograms (ECGs), enzyme replacement therapy (ERT), peripheral blood mononuclear cells (PBMC), Gait Stairs Gower Chair (GSGC) score, Motor Function Measure Scale-Short Form (MFM-20), Activity limitations for patients with upper and/or lower limb impairments (ACTIVLIM), Individualized Neuromuscular Quality of Life Questionnaire (iNQOL), maximum inspiratory pressure (MIP), sniff nasal inspiratory pressure (SNIP), maximum expiratory pressure (MEP), quick motor function test (QMFT), 12-Item Short-Form Health Survey (SF-12), Mental Component Summary (MCS), Gross Motor Function Measure-88 (GMFM-88), five-level EuroQol-5D (EQ-5D). * Number of treated LOPD patients. ** Successful desensitisation and consecutive ERT re-administration with tolerance to ERT. *** Five patients had a disease onset during childhood and one patient was diagnosed as a child. Three patients (one childhood onset and two adults) had a cardiomyopathy. **** Age at onset of 6.1–73.2 (mean ± SD 37.7 ± 15.7) years and diagnosis of 17.1–76.7 (mean ± SD 48.2 ± 14.6) years. All patients were ≥18 years of age during the study.

Author (Year)	Title	Study Design	Sample Size *	Outcome(s)
Lipinski, S.E. (2009) [33]	Desensitization of an adult patient with Pompe disease and a history of anaphylaxis to alglucosidase alfa	Case report	One patient	IgE antibodies, IgG antibodies, intradermal skin testing results, reactions during infusion, management for reactions, complement and tryptase testing
De Vries, J.M. (2010) [29]	High antibody titer in an adult with Pompe disease affects treatment with alglucosidase alfa	Case report	One patient (three reference patients)	Muscle strength (MRC, HHD), 6MWT, FVC, acid alfa-glucosidase activity in leucocytes and fibroblasts, inhibition of alglucosidase alfa uptake, IARs
Van Der Ploeg, A.T. (2010) [7]	A randomized study of alglucosidase alfa in late-onset Pompe’s disease	Randomised controlled trial	60 patients	Distance walked (6-MWT), percentage of predicted FVC, quantitative muscle testing arm (% of predicted), maximum inspiratory pressure (% of predicted), maximum expiratory pressure (% of predicted), SF36 score, antibody titre
Papadopoulos (2012) [34]	Pre-treatment antibodies against acid a-glucosidase in a patient with Late-onset Pompe disease	Case report	One patient	Antibody titre
Patel, T.T. (2012) [35]	The impact of antibodies in late-onset Pompe disease: A case series and literature review	Retrospective, cohort study, literature review	60 patients	Antibody titres, FVC in upright position, 6MWT, supine-to-stand, 4-stair climb, gait speed, MRC, SF36, physical component summary
Regnery, C (2012) [3]	36 months observational clinical study of 38 adult Pompe disease patients under alglucosidase alfa enzyme replacement therapy	Prospective, cohort study	38 patients	Walton Gardner Medwin score, arm function test, FVC, 6MWT, MRC, timed tests (modified Gowers, 10 m walk, four-stair climb), SF-36, CK levels, antibody titres, side effects of ERT
Lin (2013) [36]	Low-frequency enzyme replacement therapy in late-onset Pompe disease	Case report	One patient	FVC, 6MWT, WGM scale
Schneider, I. (2014) [37]	Enzyme replacement therapy and antibodies in late-onset Pompe disease	Cohort study	10 patients	Anti-rhGAA antibody titres, 6MWT, VC, non-invasive ventilation, wheelchair dependency
Case, L.E. (2015) [38]	Safety and efficacy of alternative alglucosidase alfa regimens in Pompe disease	Randomised, cohort study	Four patients	Treatment efficacy: GMFM-66, Pompe PEDI, MMT, ventilator use, PCS (MOSSF-36). Safety: AEs, vital signs, physical examinations, ECGs, haematology, chemistry, urinalysis and antibodies, anti-rhGAA antibody formation, inhibitory antibody formation, IgE, serum tryptase, complement activation, skin testing.
Gallay, L. (2016) [39]	SWORD: A simplified desensitization protocol for enzyme replacement therapy in adult Pompe disease	Case report	One patient	Successful ERT therapy **, IARs
Masat, E. (2016) [40]	Long-term exposure to Myozyme results in a decrease of anti-drug antibodies in late-onset Pompe disease patients	Prospective, cohort study	28 patients	Anti-rhGAA antibody titres, anti-rhGAA IgG subclasses, IgM, IgE, non-neutralising IgG, PBMC reactivity to rhGAA, T cell reactivity to rhGAA, cytokine and chemokine secretion and upregulation, FVC, 6MWT
De Vries, J.M. (2017) [41]	Pompe disease in adulthood: effects of antibody formation on enzyme replacement therapy	Prospective, cohort study	73 patients	Antibody titre, neutralising antibodies, IARs, effect of GAA variants on antibody formation, effect of antibodies on clinical outcome
Filosto, M. (2019) [42]	Assessing the Role of Anti rh-GAA in Modulating Response to ERT in a Late-Onset Pompe Disease Cohort from the Italian GSDII Study Group	Prospective, cohort study	64 patients	Antibody titre, MRC sum score, 6MWT, GSGC score, FVC
Fernandez-Simon (2019) [43]	Study of the effect of anti-rhGAA antibodies at low and intermediate titers in late onset Pompe patients treated with ERT	Prospective, cohort study	25 patients	Antibody titre, 6MWT, 10 m walk, timed up-and-go test, four-stair climb, MFM-20, MRC, HHD, ACTIVLIM, SF-36, iNQOL, muscle MRI (fat fraction)
Alandy-Dy J. (2019) [44]	Variable clinical features and genotype-phenotype correlations in 18 patients with late-onset Pompe disease	Cohort study	18 patients ***	Ambulatory status (type of assistive device being used), 6MWT, MRC scale and dynamometry, respiratory status (type of ventilatory support required), pulmonary function, FVC upright and supine, MIP, SNIP
Diaz-Manera J. (2021) [5]	Safety and efficacy of avalglucosidase alfa versus alglucosidase alfa in patients with late-onset Pompe disease (COMET): a phase 3, randomised, multicentre trial	Randomised controlled trial	49 patients ****	FVC, MIP, MEP, upper extremity muscle strength (HHD), QMFT, SF-12, PCS and MCS, gait, stairs, Gower’s Manoeuvre, chair composite score, GMFM-88, EQ-5D, Pediatric Quality of Life Inventory, anti-alglucosidase alfa antibodies and neutralising IgG antibodies, urinary hexose tetrasaccharide, serum creatine kinase, alanine aminotransferase, aspartate aminotransferase, treatment-emergent adverse events, infusion-associated reactions, Rasch-built-Pompe-specific activity scale, patient global impression of change, Pompe disease symptom scale, Pompe disease impact scale
Winkler, M. (2022) [45]	Long-term effects of enzyme replacement therapy in an elderly cohort of late-onset Pompe disease	Retrospective, cohort study	6 patients	6MWT, MRC sum score, QMFT, FVC sitting and supine, CK, anti-rhGAA IgG antibody titres, muscle biopsy

**Table 2:** **Data on a group level.** High sustained antibody titre (HSAT), infusion-associated reactions (IARs), male (M), female (F), not reported (NR), Quartile (Q), 6-Minute Walking Test (6MWT), Forced Vital Capacity (FVC), Mean Inspiratory Pressure (MIP), Mean Expiratory Pressure (MEP), 36-Item Short Form Health Survey (SF-36), Medical Research Council (MRC) sum score, creatine kinase (CK), enzyme replacement therapy (ERT), sniff nasal inspiratory pressure (SNIP), Hand Held Dynamometry (HHD), quick motor function test (QMFT), 12-Item Short-Form Health Survey (SF-12), Physical Component Summary (PCS), Mental Component Summary (MCS). * Five patients had a disease onset during childhood and one patient was diagnosed as a child. Three patients (one childhood-onset and two adults) had a cardiomyopathy. ** If dosage was not, mentioned we assumed the standard EMA/FDA-approved dosage was used. *** Age at onset of 6.1–73.2 (mean ± SD 37.7 ± 15.7) years and diagnosis of 17.1–76.7 (mean ± SD 48.2 ± 14.6) years. All patients were ≥18 years of age during the study. **** No absolute data presented; read from study figures.

Author (Year) [Ref. #]	Patients (Patients with Antibody Titres Measured)	Sex (M/F)	Age (Mean/Median)	Age Onset (Mean/Median)	Age at Diagnosis (Mean/Median)	Dosing	Treatment Duration	% of Patients with High Antibody Titre	Antibody Titre Range	Neutralising Effects Antibodies	Definition High Antibody Titre HSAT	% of Patients with IARs	Treatment Response
Regnery, C. (2012) [3]	1–38 (38)	20 M, 18 F	53.11 years average (range 27–73)	36.2 years ± 10.7 years	41.6 years (mean)	20 mg/kg/eow	36 months	5%	1:600–1:819 200	Yes, in one patient (2.6%)	Not defined	18% (7 patients)	Stable disease or improvement (Walton Gardner Medwin Score, MRC score); improved, unchanged, declined disease (timed function tests), or stable disease (SF-36); significant mean decrease in CK (improvement). Overall conclusion: stabilises natural disease course. One female discontinued ERT as a result of declining in her neuromuscular state, based on development of high anti-GAA antibody titres (1:819,000).
Diaz-Manera, J. (2021) [5]	1–49 *** (48)	25 M, 24 F	20-78 (mean 50.3 SD ± 13.7) years	6.1–73.2 (mean ± SD 37.7 ± 15.7) years	17.1–76.7 (mean ± SD 48.2 ± 14.6) years	20 mg/kg/eow	49 weeks	33% (high and persistent)	1:100–1:409,600	Yes, in four patients (8%)	≥12,800, sustained not defined	33% (16 patients)	Changes from baseline to week 49 in least-squares mean (SE). Improvements in upright FVC (% predicted) (0.46% (SE 0.93)); 6MWT (2.19 m (10.40)); 6MWT (% predicted) (0.31 (1.62)); MIP (% predicted) (4.29 (2.19)); MEP (% predicted) (8.38 (2.96)); HHD, lower extremity (153.72 (48.54)); HHD, upper extremity (109.67 (38.98)); QMFT total score (1.89 (0.69)); SF-12 PCS score (1.60 (1.07)); and SF-12 MCS (0.76 (1.32)). Effect of antibody titres on treatment outcome not assessed.
Van Der Ploeg, A.T. (2010) [7]	1–60 (59)	34 M, 26 F	NR	30.3 years (mean) ± 12.3, range 5.3–58.6	NR	20 mg/kg/eow	78 weeks	24% (supplementary data > 1:25,600)	1:200–1:819,200	18 (31%) tested positive for inhibition of enzyme uptake (out of 59)	1:25,600–819,200, Q4 titre, sustained not defined	28% (17 patients)	Significantly increased 6MWT, % pred FVC. Increase in quantitative muscle testing, MIP, MEP, and SF-36 on a group level. No consistent association was found between the serum IgG antibody titre and the coprimary efficacy end points.
Patel, T.T. (2012) [35]	1–60 (60)	NR	NR	NR	NR	20 mg/kg/eow	NR	Periodically high titres unknown, 10% out of all 60 patients had high sustained antibody titres	NR	NR	≥1:51,200 on two or more occasions at or beyond 6 months on ERT	At least 3% of the 60 patients had IARs (two out of three reported in detail)	Not reported on a group level. Effect of antibody titres on treatment outcome not assessed on a group level.
Case, L.E. (2015) [38]	1–4 (4)	2 M, 2 F	NR	NR	NR	20 mg/kg/week or 40 mg/kg/eow	52 weeks	0%	NR	None	≥1:51,200 on more than 2 occasions at or beyond 6 months on ERT	25% (1 patient)	2 patients’ motor skills improved on ERT, one patient’s motor skills maintained baseline status, one patient experienced a decline in respiratory status. Effect of antibody titres on treatment outcome not assessed.
Masat, E. (2016) [40]	1–28 (24)	13 M, 15 F	Average age 58.2 years, SD 2.4 years; median 57.5 years	NR	NR	20 mg/kg/eow	78 months on average (SD 28.8; median 84)	21% (out of 24 patients with more than 3 years on ERT)	<1:6400–≥1:25,000	Subset had elevated levels of anti-rhGAA IgG1 and IgG4 antibodies. No inhibitory activity was measured	Not defined	NR	Initial improvement or stabilisation of 6MWT and FVC measurements followed by a downward trend. No correlation was found between the evolution of FVC and 6MWT and the measurements of immune responses to rhGAA after long-term ERT
De Vries, J.M. (2017) [41]	1–73 (73)	37 M, 36 F	NR	32.1 years (1.4–62.2)	41.3 (1.4–72.7) years	20 mg/kg/eow	40 months (range 24–42 months)	22%	0 to 1:156,250	A total of 16 patients with high titres. A 15–85% neutralising effect in 8 patients with maximum titre ≥1:156,250.A 0–15% neutralising effect in patients with maximum titre 1:31,250. Only one patient evidenced interference of high sustained anti-rhGAA antibodies with ERT	≥1:31,250, sustained not defined	18% (13 patients)	In all three titre groups (none-or-low, intermediate, high), patients with improving, stable, and declining clinical courses were present. The occurrence of IARs positively correlated with the height of the titre (*p* = 0.001). The total number of IARs that a patient experienced during the study period increased with higher antibody titres (ρ = 0.46, *p* < 0.001).
Filosto, M. (2019) [42]	1–64 (64)	29 M, 35 F	median 46.45 ± 17.64 years	NR	NR	20 mg/kg/eow	4–136 months ERT	3.1%	0 to 1:31,250	NR	>1:31,250, sustained not defined	NR	Increased, stable, and decreased clinical functioning. No statistical significance was found in relating the T0–T1 delta differences and antibody titres, except for MRC sum score values in a subgroup of patients treated for <36 months, in which those with a null antibody titre showed a greater clinical improvement than patients with a positive titre.
Alandy-Dy, J. (2019) [44]	1–18 * (NR, at least 2)	14 M, 4 F	22-74 (mean ± SD 53.72 ± 14.09/median 56.50) years	5–58 (mean ± SD 29.56 ± 15.80/median 30.00) years	11–65 (mean ± SD 43.61 ± 15.82/median 44.00) years	20 mg/kg/eow **	2–11 years (up to 10.7 years after treatment initiation)	11.1%	NR	NR	Not defined	5.6% (1 patient)	Upright FVC; significant improvement in the patients’ decline after starting ERT (−0.17 per year (*p* < 0.0001)). Supine FVC; patients declined −0.55 per year after starting ERT (*p* = 0.047). Post ERT, MIP declined at 0.92 per year (*p* = 0.0169) and SNIP declined at 1.93 per year (*p* = 0.0226). Post ERT 6MWT was declining by 11.6 m per year (*p* < 0.0001). Effect of antibody titres on treatment outcome not assessed.
Winkler, M. (2022) [45]	1–6 (6)	3 M, 3 F	59–80 (median 72.5) years	40–64 (49) years	Diagnostic gap of 5–26 years (median 7.5) Age at beginning of ERT of 52–69 (median 63) years	20 mg/kg/eow	7–12 years (median 8.5)	16.6%	0 to 1:102,400 ****	NR	≥1:31,250, sustained not defined	0% (0 patients)	6MWT improved in 4/6, and 2/6 each showed an improvement or stabilisation in muscle strength and FVC supine. FVC showed a decline in all patients in a sitting position, and QMFT worsened in 5/6. CK levels decreased in all patients. Antibody titres were not associated with treatment effects. Highest titres were present in best responders who were female, still ambulatory, and without ventilatory support at follow-up.

**Table 3:** **Data on a patient level in patients with a high (as defined by the studies) maximum titre.** Data on a patient level; patients with high titres. High sustained antibody titre (HSAT), infusion-associated reactions (IARs), female (F), male (M), not reported (NR), milligram per kilogram every other week (mg/kg/eow), enzyme replacement therapy (ERT), recombinant human alpha-glucosidase (rhGAA), Medical Research Council (MRC) sum score, Forced Vital Capacity (FVC), quick motor function test (QMFT), 6-Minute Walking Test (6MWT). * Interpretation based on Pompe variant database; it should be noted that determination of the mutation based on the protein change is not 100% accurate. ** Calculated from paper data. *** If dosage was not mentioned, we assumed the standard EMA/FDA-approved dosage was used. **** No absolute data presented; read from study figures. ^#^ Same patient.

Author (Year) [Ref. #]	Patient No.	Sex	Age	Allele 1, Protein Change	Allele 2, Protein Change	Age at Onset	Age at Diagnosis	Age at Start of Treatment	Dosing	Treatment Duration	Highest Antibody Titre (Neutralising Effect)	Definition High Antibody Titre and High Sustained Antibody Titre (HSAT)	IARs	Treatment Response
De Vries, J.M. (2010) [29]	1 #	M	NR	c. -32-13T>G p.[=,0] *	c.1548G>A * p.Trp516X	29 years	39 years	50 years	20 mg/kg/eow	35 months	1:800,000 (42% of alglucosidase alfa captured by antibodies, uptake inhibited)	Not defined	≥1	Poor; declining clinical parameters on ERT
Papadopoulos, C (2012) [34]	1	F	37	c.-32-13T>G p.[=,0] *	c.2066_2070dup p.(Ala691Serfs*7) *	NR	NR	37 years	20 mg/kg/eow ***	24 weeks	1:102,400 (no neutralising antibodies)	Not defined	None mentioned	NR
Patel, T.T. (2012) [35]	1	M	NR	c.-32-13T>G p.[=,0] *	c.2238G>A p.(Trp746*) *	32 years	28 years	37 years	20 mg/kg/eow	255 weeks	1:102,400 (NR)	≥1:51,200 on two or more occasions at or beyond 6 months on ERT	No significant ones	Clinical decline
Patel, T.T. (2012) [35]	2	F	NR	c.-32-13T>G p.[=,0] *	c.1075G>A p.[(Gly359Arg) (Val358Aspfs*33)] *	41 years	49 years	56 years	20 mg/kg/eow	202 weeks	1:204,800 (NR)		≥1	Initial stable disease; clinical decline after 54 weeks
Patel, T.T. (2012) [35]	3	F	NR	c.-32-13T>G p.[=,0] *	c.1076-22T>G p.? *	25 years	41 years	57 years	20 mg/kg/eow	68 weeks	1:819,200 (NR)		≥1	Initial improvement or stable disease until week 32; rapid clinical decline at 60 weeks
De Vries, J.M. (2017) [41]	1 #	NR	NR	NR	NR	NR	NR	NR	20 mg/kg/eow	NR	1:3,906,250 (strong neutralising effects)	≥1:31,250, sustained not defined	NR	Clear interference of ERT by anti-rhGAA antibodies: decline in MRC and FVC scores
De Vries, J.M. (2017) [41]	2	NR	NR	NR	NR	NR	NR	NR	20 mg/kg/eow	NR	≥156,250 (strong neutralising effects)		NR	Unclear due to high, stable MRC scores and FVC
De Vries, J.M. (2017) [41]	3	NR	NR	NR	NR	NR	NR	NR	20 mg/kg/eow	NR	≥156,250 (strong neutralising effects)		NR	Unclear due to high, stable MRC scores and FVC
De Vries, J.M. (2017) [41]	4	NR	NR	NR	NR	NR	NR	NR	20 mg/kg/eow	NR	≥156,250 (strong neutralising effects)		NR	Unclear due to high, stable MRC scores and FVC
De Vries, J.M. (2017) [41]	5	NR	NR	N	NR	NR	NR	NR	20 mg/kg/eow	NR	≥156,250 (temporary neutralising effects)		NR	No effect of antibodies
De Vries, J.M. (2017) [41]	6	NR	NR	NR	NR	NR	NR	NR	20 mg/kg/eow	NR	≥156,250 (temporary neutralising effects)		NR	No effect of antibodies
De Vries, J.M. (2017) [41]	7	NR	NR	NR	NR	NR	NR	NR	20 mg/kg/eow	NR	≥156,250 (temporary neutralising effects)		NR	No effect of antibodies
De Vries, J.M. (2017) [41]	8	NR	NR	NR	NR	NR	NR	NR	20 mg/kg/eow	NR	≥156,250 (no neutralising effects)		NR	No effect of antibodies
Fernandez-Simon, E. (2019) [43]	15	F	65	c.1781G>A p.(Arg594His) *	c. 1194+5G>A p.? *	NR	NR	64 **	20 mg/kg/eow	NR	1:51,200 (NR)	>1:31,200, sustained not defined	NR	Results of muscle function tests and spirometry remained stable after one year of progression in this patient, and there were not significant differences in mean thighs fat fraction
Alandy-Dy, J. (2019) [44]	9	F	74	c.-32-13T>G p.[=,0] *	c.2655_2656delCG p.(Val886Glufs*2) *	30s	62	63	20 mg/kg/eow ***	11 years **	1:12,800 (NR)	Not defined	≥1	NR
Alandy-Dy, J. (2019) [44]	10	M	70	c.-32-13T>G p.[=,0] *	c.2655_2656delCG p.(Val886Glufs*2) *	58	65	66	20 mg/kg/eow ***	4 years **	High antibody titre (NR)	Not defined	None	NR
Winkler, M. (2022) [45]	6	F	NR	c.-32-13T>G p.[=,0] *	NR	Not reported	Not reported	Not reported	20 mg/kg/eow	Not reported	1:102,400 (NR) ****	≥1:31,250, sustained not defined	None	MRC improvement followed by stabilisation, QMFT improvement, 6MWT improvement, FVC sitting stabilisation followed by decline, FVC supine improvement

## Supplementary Materials

The following supporting information can be downloaded at: https://www.mdpi.com/article/10.3390/biom13091414/s1.
