# Supplementary material for: Are Anti-rhGAA Antibodies a Determinant of Treatment Outcome in Adults with Late-Onset Pompe Disease? A Systematic Review
Source: Biomolecules. 2023 Sep 19;13(9):1414. doi: 10.3390/biom13091414 (PMC10526476; doi:10.3390/biom13091414)
Supplement: Supplementary file 1 [file biomolecules-13-01414-s001.zip › Supplementary File S1.pdf]

Supplementary File S1

Initial literature search (12-03-2019)

Database	*# of refs*	*After de-duplication*
Embase.com	1441	1375
Medline Ovid	697	70
Web of Science	1136	377
PsycInfo Ovid	65	7
Cochrane (Clinical Trials only)	45	11
Google Scholar (random top-200)	139	43
**Total**	**3523**	**1883**

Embase – 1441 refs
('glycogen storage disease type 2'/exp OR (pompe* OR ((glycogen*) NEAR/3 (storage*) NEAR/3 (2 OR ii)) OR ((glucosidas*) NEAR/3 (syndrom* OR diseas* OR deficien*)) OR ((diffuse* OR 2 OR ii) NEAR/3 (glycogenos*)) OR acid-maltas* OR GSD-2 OR GSD2 OR GSD-ii OR GSDii):ab,ti) **AND** ((juvenil* OR late OR later OR adult* OR ((non) NEAR/3 (infantil*))):ab,ti) ***NOT*** *([animals]/lim NOT [humans]/lim)* ***AND*** *[1999-2019]/py*

Medline – 697 refs *(limited in database to publication year 1999-2019)*
(Glycogen Storage Disease Type II/ OR (pompe* OR ((glycogen*) ADJ3 (storage*) ADJ3 (2 OR ii)) OR ((glucosidas*) ADJ3 (syndrom* OR diseas* OR deficien*)) OR ((diffuse* OR 2 OR ii) ADJ3 (glycogenos*)) OR acid-maltas* OR GSD-2 OR GSD2 OR GSD-ii OR GSDii).ab,ti.) **AND** ((juvenil* OR late OR later OR adult* OR ((non) ADJ3 (infantil*))).ab,ti.) ***NOT*** *(exp animals/ NOT humans/)*

PsycINFO – 65 refs *(limited in database to publication year 1999-2019)*
((pompe* OR ((glycogen*) ADJ3 (storage*) ADJ3 (2 OR ii)) OR ((glucosidas*) ADJ3 (syndrom* OR diseas* OR deficien*)) OR ((diffuse* OR 2 OR ii) ADJ3 (glycogenos*)) OR acid-maltas* OR GSD-2 OR GSD2 OR GSD-ii OR GSDii).ab,ti.) **AND** ((juvenil* OR late OR later OR adult* OR ((non) ADJ3 (infantil*))).ab,ti.)

Cochrane (RCTs) – 45 refs *(no articles found with a publication year before 1999)*
((pompe* OR ((glycogen*) NEAR/3 (storage*) NEAR/3 (2 OR ii)) OR ((glucosidas*) NEAR/3 (syndrom* OR diseas* OR deficien*)) OR ((diffuse* OR 2 OR ii) NEAR/3 (glycogenos*)) OR (acid NEXT/1 maltas*) OR GSD-2 OR GSD2 OR GSD-ii OR GSDii):ab,ti) **AND** ((juvenil* OR late OR later OR adult* OR ((non) NEAR/3 (infantil*))):ab,ti)

Web of Science – 1136 refs *(limited in database to publication year 1999-2019)*
TS=(((pompe* OR ((glycogen*) NEAR/2 (storage*) NEAR/2 (2 OR ii)) OR ((glucosidas*) NEAR/2 (syndrom* OR diseas* OR deficien*)) OR ((diffuse* OR 2 OR ii) NEAR/2 (glycogenos*)) OR acid-

maltas* OR GSD-2 OR GSD2 OR GSD-ii OR GSDii)) **AND** ((juvenil* OR late OR later OR adult* OR ((non) NEAR/2 (infantil*)))) *NOT ((animal* OR rat OR rats OR mouse OR mice OR murine OR dog OR dogs OR canine OR cat OR cats OR feline OR rabbit OR cow OR cows OR bovine OR rodent* OR sheep OR ovine OR pig OR swine OR porcine OR veterinar* OR chick* OR zebrafish* OR baboon* OR nonhuman* OR primate* OR cattle* OR goose OR geese OR duck OR macaque* OR avian* OR bird* OR fish*) NOT (human* OR patient* OR women OR woman OR men OR man)))*

Google Scholar – (random top-200) – 139 refs *(articles with a publication year before 1999 manually deleted in EndNote)*
pompe|"glycogen storage 2|ii"|glucosidase syndrome|disease|deficiency"|"diffuse|2|ii glycogenose"|acid-maltase juvenile|late|later|adult

<u>Updated literature search (21-06-2022)</u>

Database searched	Platform	Years of coverage	Records	Records after duplicates removed
Embase	Embase.com	1971 - Present	1945	1855
Medline ALL	Ovid	1946 - Present	935	86
Web of Science Core Collection*	Web of Knowledge	1975 - Present	1516	461
Cochrane Central Register of Controlled Trials	Wiley	1992 - Present	113	46
PsycINFO	Ovid	1806 - Present	72	14
Additional Search Engines: Google Scholar			193	36
**Total**			**4774**	**2498**

New references in EndNote file: 679

Embase
('glycogen storage disease type 2'/exp OR (pompe* OR ((glycogen*) NEAR/3 (storage*) NEAR/3 (2 OR ii)) OR ((glucosidas*) NEAR/3 (syndrom* OR diseas* OR deficien*)) OR ((diffuse* OR 2 OR ii) NEAR/3 (glycogenos*)) OR acid-maltas* OR GSD-2 OR GSD2 OR GSD-ii OR GSDii):ab,ti,kw) **AND** ((juvenil* OR late OR later OR adult* OR ((non) NEAR/3 (infantil*))):ab,ti,kw) ***NOT*** *([animals]/lim NOT [humans]/lim)* ***AND*** *[1999-2022]/py*

Medline – *(limited in database to publication year 1999-2022)*
(Glycogen Storage Disease Type II/ OR (pompe* OR ((glycogen*) ADJ3 (storage*) ADJ3 (2 OR ii)) OR ((glucosidas*) ADJ3 (syndrom* OR diseas* OR deficien*)) OR ((diffuse* OR 2 OR ii) ADJ3

(glycogenos*)) OR acid-maltas* OR GSD-2 OR GSD2 OR GSD-ii OR GSDii).ab,ti,kf.) **AND** ((juvenil* OR late OR later OR adult* OR ((non) ADJ3 (infantil*))).ab,ti,kf.) ***NOT*** *(exp animals/ NOT humans/)*

PsycINFO – *(limited in database to publication year 1999-2022)*
((pompe* OR ((glycogen*) ADJ3 (storage*) ADJ3 (2 OR ii)) OR ((glucosidas*) ADJ3 (syndrom* OR diseas* OR deficien*)) OR ((diffuse* OR 2 OR ii) ADJ3 (glycogenos*)) OR acid-maltas* OR GSD-2 OR GSD2 OR GSD-ii OR GSDii).ab,ti.) **AND** ((juvenil* OR late OR later OR adult* OR ((non) ADJ3 (infantil*))).ab,ti.)

Cochrane (RCTs) *(articles with a publication year before 1999 manually deleted in EndNote)*
((pompe* OR ((glycogen*) NEAR/3 (storage*) NEAR/3 (2 OR ii)) OR ((glucosidas*) NEAR/3 (syndrom* OR diseas* OR deficien*)) OR ((diffuse* OR 2 OR ii) NEAR/3 (glycogenos*)) OR (acid NEXT/1 maltas*) OR GSD-2 OR GSD2 OR GSD-ii OR GSDii):ab,ti,kw) **AND** ((juvenil* OR late OR later OR adult* OR ((non) NEAR/3 (infantil*))):ab,ti,kw)

Web of Science
TS=(((pompe* OR ((glycogen*) NEAR/2 (storage*) NEAR/2 (2 OR ii)) OR ((glucosidas*) NEAR/2 (syndrom* OR diseas* OR deficien*)) OR ((diffuse* OR 2 OR ii) NEAR/2 (glycogenos*)) OR acid-maltas* OR GSD-2 OR GSD2 OR GSD-ii OR GSDii)) **AND** ((juvenil* OR late OR later OR adult* OR ((non) NEAR/2 (infantil*)))) *NOT ((animal* OR rat OR rats OR mouse OR mice OR murine OR dog OR dogs OR canine OR cat OR cats OR feline OR rabbit OR cow OR cows OR bovine OR rodent* OR sheep OR ovine OR pig OR swine OR porcine OR veterinar* OR chick* OR zebrafish* OR baboon* OR nonhuman* OR primate* OR cattle* OR goose OR geese OR duck OR macaque* OR avian* OR bird* OR fish*) NOT (human* OR patient* OR women OR woman OR men OR man)))* AND PY=(1999-2022)

Google Scholar – (top-200 relevance rank) – 193 refs *(articles with a publication year before 1999 manually deleted in EndNote)*
pompe|"glycogen storage 2|ii"|glucosidase syndrome|disease|deficiency"|"diffuse|2|ii glycogenose"|acid-maltase juvenile|late|later|adult
