# Supplementary material for: Are Anti-rhGAA Antibodies a Determinant of Treatment Outcome in Adults with Late-Onset Pompe Disease? A Systematic Review
Source: Biomolecules. 2023 Sep 19;13(9):1414. doi: 10.3390/biom13091414 (PMC10526476; doi:10.3390/biom13091414)
Supplement: Supplementary file 1 [file biomolecules-13-01414-s001.zip › Supplementary Table S1.pdf]

Author (year) [Ref. #]	Patient no.	Sex	Age	Allele 1, protein change	Allele 2, protein change	Age at onset	Age at diagnosis	Age at start treatment	Dosing	Treatment duration	Highest antibody titer (neutralising effect)	Definition high antibody titer and high sustained antibody titer (HSAT)	IARs	Treatment response
De Vries, J.M. (2010) [29]	2	M	NR	c. -32-13T>G, p.[=,0]*	c.925G>A * p.Gly309Arg	46	52	54	20mg/kg/eow	NR	0 (NR)	Not defined	None	Good response
De Vries, J.M. (2010) [29]	3	F	NR	c. -32-13T>G p.[=,0]*	Not in Pompe variant database p.Glu176fs	36	62	63	20mg/kg/eow	NR	0 (NR)		None	Good response
De Vries, J.M. (2010) [29]	4	F	NR	c. -32-13T>G p.[=,0]*	Not in Pompe variant database p.Cys127fs	44	51	54	20mg/kg/eow	NR	0 (NR)		None	Good response
Lipinski, S.E. (2009) [33]	1	F	NR	NR	NR	NR	28	NR	10mg/kg/w, 20mg/kg/eow, 10 mg/kg/w	13 weeks, interpretation of data	1:1,600 (NR)	Not defined	7	NR
Lin, D.S. (2013) [36]	1	F	37	NR	NR	NR	26	33	20mg/kg/eow, 20 mg/kg every 4 weeks	4 years	NR[#]	Not defined	NR	Good, pulmonary and motor function remained stable, respiratory function and endurance responded well during year one and maintained stable afterwards
Schneider, I. (2014) [37]	1	M	52 (start observation)	c.-32-13T>G p.[=,0]*	c.307T>G * p.C103G	NR	NR	48 **	20mg/kg/eow***	4 years in study	0 (NR)	> 1: 51,200 on two or more occasions at or beyond 6 months on ERT	NR	Stable disease course under ERT
Schneider, I. (2014) [37]	2	M	44 (start observation)	c.-32-13T>G p.[=,0]*	c.1495T>A * p.W499R	NR	NR	40 **	20mg/kg/eow***	4 years in study	1:25,600 (NR)		NR	Antibody titer of 1:25,600 had no impact on the functional status
Schneider, I. (2014) [37]	3	F	69 (start observation)	c.-32-13T>G p.[=,0]*	c.2481+102_2646+31 del p.(Gly828_Asn882del) *	NR	NR	66 **	20mg/kg/eow***	4 years in study	1:3,200 (NR)		NR	Stable disease course under ERT
Schneider, I. (2014) [37]	4	M	43 (start observation)	c.-32-13T>G p.[=,0]*	c.2136_2137del p.(Phe713Profs*23) *	NR	NR	41 **	20mg/kg/eow***	4 years in study	1:6,400 (NR)		NR	Stable disease course under ERT
Schneider, I. (2014) [37]	5	F	36 (start observation)	c.1478C>T * p.P493L	c.1655T>C * p.L552P	NR	NR	34 **	20mg/kg/eow***	4 years in study	1:3,200 (NR)		NR	Stable disease course under ERT
Schneider, I. (2014) [37]	6	F	47 (start observation)	c.-32-13T>G p.[=,0]*	c.1655T>C * p.L552P	NR	NR	45 **	20mg/kg/eow***	4 years in study	1:25,600 (NR)		NR	Decline at 40 months of ERT, need for non-invasive ventilation and walking aids
Schneider, I. (2014) [37]	7	M	63 (start observation)	c.-32-13T>G p.[=,0]*	c.925G>A * p.G309R	NR	NR	63 **	20mg/kg/eow***	4 years in study	1:400 (NR)		NR	Stable disease course under ERT
Schneider, I. (2014) [37]	8	F	19 (start observation)	c.-32-13T>G p.[=,0]*	c.1438-1G>C p.? *	NR	NR	19 **	20mg/kg/eow***	4 years in study	1:200 (NR)		NR	Stable disease course under ERT
Schneider, I. (2014) [37]	9	F	56 (start observation)	c.307T>G * p.C103G	c.1478C>T * p.P493L	NR	NR	56 **	20mg/kg/eow***	4 years in study	<100 (NR)		NR	Stable disease course under ERT
Schneider, I. (2014) [37]	10	M	47 (start observation)	c.307T>G * p.C103G	c.1478C>T * p.P493L	NR	NR	47 **	20mg/kg/eow***	4 years in study	1:1,600 (NR)		NR	Stable disease course under ERT
Gallay, L. (2016) [39]	1	F	NR	c. 1_15T>G Not in Pompe variant database	c.1636+1G>C p.? *	42	44	44	20mg/kg/eow	3 years	0[##] (NR)	Not defined	≥ 1	Significant efficiency regarding recent improvement of clinical test and respiratory investigations
Fernandez-Simon, E. (2019) [43]	1	F	50	c.-32-13T>G p.[=,0]*	c.1076-1G>C p.? *	NR	NR	47 **	20mg/kg/eow	NR	1:800 (NR)	>1:31,200, sustained not defined	NR	NR
Fernandez-Simon, E. (2019) [43]	2	F	48	c.-32-13T>G p.[=,0]*	c.2173C>T p.(Arg725Trp) *	NR	NR	39 **	20mg/kg/eow	NR	1:1,600 (NR)		NR	NR
Fernandez-Simon (2019) [43}	3	F	63	c.-32-13T>G p.[=,0]*	c.2600_2604delinsA p.(Val867Glufs*19) *	NR	NR	59 **	20mg/kg/eow	NR	0 (NR)		NR	NR
Fernandez-Simon, E. (2019) [43]	4	F	45	c.-32-13T>G p.[=,0]*	c.1532C>A Not in Pompe variant database	NR	NR	42 **	20mg/kg/eow	NR	1:3,200 (NR)		NR	NR
Fernandez-Simon, E. (2019) [43]	5	F	51	c.-32-13T>G p.[=,0]*	c.236_246del p.(Pro79Argfs*13) *	NR	NR	47 **	20mg/kg/eow	NR	1:1,600 (NR)		NR	NR

Fernandez-Simon, E. (2019) [43]	6	M	66	c.-32-13T>G p.[=,0]*	c. 1933G>T p.(Asp645Tyr)*	NR	NR	66 **	20mg/kg/eow	NR	0 (NR)		NR	NR
Fernandez-Simon, E. (2019) [43]	7	F	59	c.-32-13T>G p.[=,0]*	c.1637G>A Not in Pompe variant database	NR	NR	52 **	20mg/kg/eow	NR	1:1,600 (NR)		NR	NR
Fernandez-Simon, E. (2019) [43]	8	F	55	c.-32-13T>G p.[=,0]*	c.2173C>T p.(Arg725Trp) *	NR	NR	48 **	20mg/kg/eow	NR	0 (NR)		NR	NR
Fernandez-Simon (2019 [43])	9	M	42	c.-32-13T>G p.[=,0]*	c.573C>A p.(Tyr191*) *	NR	NR	39 **	20mg/kg/eow	NR	1:6,400 (NR)		NR	NR
Fernandez-Simon, E. (2019) [43]	10	F	31	c.-32-13T>G p.[=,0]*	c.1637G>A Not in Pompe variant database	NR	NR	26 **	20mg/kg/eow	NR	1:3,200 (NR)		NR	NR
Fernandez-Simon, E. (2019) [43]	11	F	39	c.-32-13T>G p.[=,0]*	c. 1655T>C p.(Leu552Pro) *	NR	NR	32 **	20mg/kg/eow	NR	1:3,200 (NR)		NR	NR
Fernandez-Simon, E. (2019) [43]	12	M	47	c.2173C>T p.(Arg725Trp) *	c.2173C>T p.(Arg725Trp) *	NR	NR	45 **	20mg/kg/eow	NR	1:6,400 (NR)		NR	NR
Fernandez-Simon, E. (2019) [43]	13	M	51	c.-32-13T>G p.[=,0]*	c.1657C>T p.(Gln553*) *	NR	NR	45 **	20mg/kg/eow	NR	1:1,600 (NR)		NR	NR
Fernandez-Simon, E. (2019) [43]	14	F	51	c.-32-13T>G p.[=,0]*	c.1657C>T p.(Gln553*) *	NR	NR	46 **	20mg/kg/eow	NR	0 (NR)		NR	NR
Fernandez-Simon, E. (2019) [43]	16	F	35	c.-32-13T>G p.[=,0]*	c.1A>T p.(0) *	NR	NR	29 **	20mg/kg/eow	NR	1:3,200 (NR)		NR	NR
Fernandez-Simon, E. (2019) [43]	17	F	40	c.-32-13T>G p.[=,0]*	c.1889-1G>A Not in Pompe variant database	NR	NR	39 **	20mg/kg/eow	NR	0 (NR)		NR	NR
Fernandez-Simon, E. (2019) [43]	18	F	52	c.1781G>A p.(Arg594His) *	c.1194+5G>A p.? *	NR	NR	45 **	20mg/kg/eow	NR	1:1,600 (NR)		NR	NR
Fernandez-Simon, E. (2019) [43]	19	M	64	c.-32-13T>G p.[=,0]*	c.2481+102_2646+31 del p.(Gly828_Asn882del) *	NR	NR	62 **	20mg/kg/eow	NR	0 (NR)		NR	NR
Fernandez-Simon, E. (2019) [43]	20	F	57	c.-32-13T>G p.[=,0]*	c. 1447G>T p.(Gly483Trp) *	NR	NR	54 **	20mg/kg/eow	NR	1:12,800 (NR)		NR	NR
Fernandez-Simon, E. (2019) [43]	21	M	46	c.-32-13T>G p.[=,0]*	c. 1532C>A Not in Pompe variant database	NR	NR	45 **	20mg/kg/eow	NR	1:6,400 (NR)		NR	NR
Fernandez-Simon, E. (2019) [43]	22	M	51	c.-32-13T>G p.[=,0]*	c. 1933G>T p.(Asp645Tyr) *	NR	NR	50 **	20mg/kg/eow	NR	1:1,600 (NR)		NR	NR
Fernandez-Simon, E. (2019) [43]	23	M	43	c.-32-13T>G p.[=,0]*	c. 1408_1410delinsTTT Not in Pompe variant database	NR	NR	42 **	20mg/kg/eow	NR	1:1,600 (NR)		NR	NR
Fernandez-Simon, E. (2019) [43]	24	F	51	c.-32-13T>G p.[=,0]*	c.-32-13T>G p.[=,0] *	NR	NR	45 **	20mg/kg/eow	NR	1:1,600 (NR)		NR	NR
Fernandez-Simon, E. (2019) [43]	25	M	42	c.-32-13T>G p.[=,0]*	c. 655G>A p.(Gly219Arg) *	NR	NR	42 **	20mg/kg/eow	NR	0 (NR)		NR	NR

Alandy-Dy, J. (2019) [44]	2	M	48	c.1935C>A p.(Asp645Glu)*	c.1594G>A p.(Gly532Ser)*	39	43	44	20mg/kg/eow***	4 years**	NR		None	NR
Alandy-Dy, J. (2019) [44]	3	M	56	c.-32-13T>G p.[=,0]*	c.2431delC p.(Leu811Trpfs*37)*	30s	48	48	20mg/kg/eow***	8 years**	NR		None	NR
Alandy-Dy, J. (2019) [44]	6	F	57	c.-32-13T>G p.[=,0]*	c.925G>A p.(Gly309Arg)*	20s	31	46	20mg/kg/eow***	11 years**	NR		None	NR
Alandy-Dy, J. (2019) [44]	7	M	50	c.-32-13T>G p.[=,0]*	c.925G>A p.(Gly309Arg)*	32	38	43	20mg/kg/eow***	7 years**	NR		None	NR
Alandy-Dy, J. (2019) [44]	8	F	49	c.-32-13T>G p.[=,0]*	c.1437+2T>C p.(Asp443_Lys479del)*	20s	42	42	20mg/kg/eow***	7 years**	NR		None	NR
Alandy-Dy, J. (2019) [44]	11	M	68	c.-32-13T>G p.[=,0]*	c.1951-1952delGGinsT p.(Gly651Serfs*45)*	30s	55	61	20mg/kg/eow***	7 years**	NR	Not defined	None	NR
Alandy-Dy, J. (2019) [44]	12	M	63	c.-32-13T>G p.[=,0]*	c.1951-1952delGGinsT p.(Gly651Serfs*45)*	35	55	56	20mg/kg/eow***	7 years**	NR		None	NR
Alandy-Dy, J. (2019) [44]	13	M	50	c.-32-13T>G p.[=,0]*	c.525_526delTG p.(Asn177Profs*11)*	42	45	45	20mg/kg/eow***	5 years**	NR		None	NR
Alandy-Dy, J. (2019) [44]	14	M	63	c.-32-13T>G p.[=,0]*	c.1445C>G p.(Pro482Arg)*	47	58	58	20mg/kg/eow***	5 years**	NR		None	NR
Alandy-Dy, J. (2019) [44]	16	M	64	c.-32-13T>G p.[=,0]*	c.307T>G p.(Cys103Gly)*	56	60	61	20mg/kg/eow***	3 years**	NR		None	NR
Alandy-Dy, J. (2019) [44]	18	M	65	c.-32-13T>G p.[=,0]*	c.1051delG p.(Val351Cysfs*41)*	40	62	62	20mg/kg/eow***	3 years**	NR		None	NR
Winkler, M. (2022) [45]	1	NR	NR	c.-32-13T>G p.[=,0]*	NR	NR	NR	NR	20mg/kg/eow	NR	1:3,200 (NR)****		None	MRC stabilization followed by decline, QMFT decline, 6MWT decline followed by improvement, FVC sitting and supine decline
Winkler, M. (2022) [45]	2	NR	NR	c.-32-13T>G p.[=,0]*	NR	NR	NR	NR	20mg/kg/eow	NR	0 (NR)		None	MRC stabilization, QMFT decline, 6MWT decline, FVC sitting improvement followed by decline, FVC supine stabilization
Winkler, M. (2022) [45]	3	NR	NR	c.-32-13T>G p.[=,0]*	NR	NR	NR	NR	20mg/kg/eow	NR	1:800 (NR)****	≥1:31,250, sustained not defined	None	MRC improvement followed by decline, QMFT decline, 6MWT improvement followed by decline, FVC sitting and supine stabilization followed by decline
Winkler, M. (2022) [45]	4	NR	NR	c.-32-13T>G p.[=,0]*	NR	NR	NR	NR	20mg/kg/eow	NR	1:25,600 (NR)****		None	MRC decline followed by stabilization, QMFT decline, 6MWT improvement, FVC sitting and supine decline
Winkler, M. (2022) [45]	5	NR	NR	c.-32-13T>G p.[=,0]*	NR	NR	NR	NR	20mg/kg/eow	NR	1:1,600 (NR)****		None	MRC stabilization, QMFT stabilization followed by decline, 6MWT decline, FVC sitting and supine decline

Supplementary Table S1: Data on a patient level in patients with a low to intermediate(as defined by the studies) maximum titer. *High Sustained Antibody Titer (HSAT), Infusion Associated Reactions (IARs), milligram per kilogram every other week (mg/kg/eow), F (F), Male (M), Enzyme Replacement Therapy (ERT), milligram per kilogram every other week (mg/kg/eow), Not reported (NR), Enzyme Replacement Therapy (ERT), recombinant human alpha-Glucosidase (rhGAA), Medical Research Council (MRC) sum score, quick motor function test (QMFT), 6 Minute walking test (6MWT), Forced Vital Capacity (FVC). *Interpretation based on Pompe variant database, it should be noted that in case of determination of the mutation based on the protein change is not 100% accurate. **Calculated from paper data. *** If dosage is not mentioned we assumed the standard EMA/FDA approved dosage was used. **** No absolute data presented, read from study figures. [#]Anti-rhGAA IgG antibody titer was NR in this study, this study was included because it reports on alternative dosing. [##]IgE antibody titer.*
